# C14orf166 overexpression correlates with tumor progression and poor prognosis of breast cancer

**DOI:** 10.1186/s12967-016-0805-0

**Published:** 2016-02-17

**Authors:** Tuck-yun Cheang, Hong-yan Zhou, Wei Chen, Bing Zhang, Liangshuai Liu, Jianyong Yang, Shenming Wang, Heping Li

**Affiliations:** Department of Thyroid and Breast Surgery, The First Affiliated Hospital of Sun Yat-sen University, 510080 Guangzhou, People’s Republic of China; Department of Neurological Intensive Care Unit, The First Affiliated Hospital of Sun Yat-sen University, 510080 Guangzhou, People’s Republic of China; Department of Medical Imaging, The First Affiliated Hospital of Sun Yat-sen University, 510080 Guangzhou, People’s Republic of China; Department of Medical Oncology, The First Affiliated Hospital of Sun Yat-sen University, 510080 Guangzhou, People’s Republic of China

**Keywords:** Breast cancer, C14orf166, Prognostic factor, Cell proliferation

## Abstract

**Background:**

Chromosome 14 open reading frame 166 (C14orf166) is upregulated in various tumors, but its role in breast cancer has not been reported.

**Methods:**

Quantitative real-time PCR and western blot were used to determine C14orf166 expression in normal breast epithelial cells (NBEC), breast cancer cells, and four matched pairs of breast cancer tissues and adjacent noncancerous tissues. Using immunohistochemistry, we determined C14orf166 expression in paraffin-embedded tissues from 121 breast cancer patients. Statistical analyses were performed to examine the associations among C14or166 expression, clinicopathological parameters and prognosis outcome of breast cancer. MTT and colony formation assay were used to determine the effect of C14orf166 on cell proliferation by overexpression or knockdown of C14orf166 level.

**Results:**

C14orf166 was upregulated in breast cancer cell lines and tissues compared with the normal cells and adjacent normal breast tissues, high C14orf166 expression was positively with advancing clinical stage. The correlation analysis between C14orf166 expression and clinicopathological characteristics suggested C14orf166 expression was significantly correlated with clinical stages, T classification, N classification and PR expression, Kaplan–Meier curves with log rank tests showed patients with low C14orf166 expression had better survival, Cox-regression analysis suggested C14orf166 was an unfavorable prognostic factor for breast cancer patients. C14orf166 overexpression promoted breast cancer cell proliferation, whereas knockdown of C14orf166 inhibited this effect. Further analysis found C14orf166 overexpression inhibited cell cycle inhibitors P21 and P27 expression, and increased the levels of Cyclin D1 and phosphorylation of Rb, suggesting C14orf166 contributed to cell proliferation by regulating G1/S transition.

**Conclusion:**

Our findings suggested C14orf166 could be a novel prognostic biomarker of breast cancer, it also contributes to cell proliferation by regulating G1/S transition.

**Electronic supplementary material:**

The online version of this article (doi:10.1186/s12967-016-0805-0) contains supplementary material, which is available to authorized users.

## Background

Breast cancer is one of the leading causes of cancer death in women worldwide [1]. It is a heterogeneous tumor, heterogeneity exists between and within subtypes of breast cancer cells [2]. The subtype of breast cancer can be divided based on the expression of estrogen receptor (ER), progesterone receptor (PR) and Her2 receptor tyrosine kinase (Her2). Thereinto,“triple negative”(ER^−^/PR^−^/Her2^−^) breast cancer is the most aggressive and has poorest prognosis [3]. Some drugs have been used to treat breast cancer, for example, Sunitinib [4], Bevacizumab [5] and Trastuzumab [6]. But breast cancer have generated resistance to some drugs, for example, Her2^+^ breast cancer have generated resistance to Trastumab [7]. The prevention and treatment are important for breast cancer, so it’s essential to find novel prognostic factors and targets for breast cancer.

C14orf166 plays critical role in transcription initiation, HCV infection, RNA metabolism, and centrosome architecture. It can interact with PA subunit of influenza virus polymerase to activate some genes transcription and promote influenza virus replication [8]. Knockdown of C14orf166 suppresses the synthesis of about 50 % RNA polymerase II transcripts, these suggest C14orf166 regulates gene transcription [9]. Junwei Lee and colleagues use proteomics approach find that C14orf166 also interacts with HCVc174 which a mature hepatitis C virus core protein, suggesting it may regulate HCV or cellular function during HCV infection [10]. C14orf166 interacts with DDX1, HSPC117 and FAM98B to form a complex to transport RNAs between the nucleus and the cytoplasm, suggesting it regulates the fate of RNA [11]. C14orf166 blocks the phosphorylation of ninein to regulation the function of ninein, ninein is required for the centrosome maturation, this suggests C14orf166 may participate in the centrosome architecture [12, 13].

C14orf166 is overexpressed in many tumors, such as pancreatic adenocarcinoma, brain tumor [13] and nasopharyngeal carcinoma. In nasopharyngeal carcinoma, C14or166 is overexpressed in nasopharyngeal carcinoma tissues, its expression is significantly correlated with gender, distant metastasis, vital status, T classification, N classification and clinical stage. It is an unfavorable prognostic factor for nasopharyngeal carcinoma patients [14]. In pancreatic adenocarcinoma, C14orf166 is overexpressed in the serum of patients with pancreatic adenocarcinoma detected by surface enhanced laser desorption/ionization time-of-flight mass spectrometry (SELDI-TOF–MS), and is a potential biomarker for pancreatic adenocarcinoma [15]. But the role of C14orf166 in breast cancer hasn’t been studied.

Here, we determined C14orf166 expression in breast cancer cells and tissues used quantitative real-time PCR and western blot. Then we determined C14orf166 expression of a cohort of 121 breast cancer patients, and examined the relationship between C14orf166 expression and clinicopathological characteristic and clinical outcome. Finally, we determined the effect of C14orf166 on cell proliferation by modulating its expression. We found C14orf166 not only was a valuable prognostic factor for patients with breast cancer, but also promoted breast cancer proliferation.

## Methods

### Human breast cancer tissue specimens

Four fresh breast cancer tissues and their adjacent non-cancerous breast tissues were obtained from the First Affiliated Hospital, Sun Yat-Sen University, China, and frozen and stored in liquid nitrogen for further use. A cohort of 121 formalin-fixed and paraffin-embedded primary breast cancer samples also were obtained from the First Affiliated Hospital, Sun Yat-Sen University, China, from 2001 to 2008. All samples were confirmed by histological and clinical diagnosis. For the use of these clinical samples for research purposes, all samples obtained with written informed and approved by the Institutional Research Ethics Committee of the First Affiliated Hospital of Sun Yat-Sen University. The detailed clinical information about these samples is shown in Additional file 1: Table S1. The clinical stages of all the patients were classified according to the 2002 TNM staging of UICC (International Union against Cancer).

### Cell culture and transfection

Normal human breast epithelial cells (NBEC) were cultured in Keratinocyte serum-free medium (Life Technologies) supplemented with epithelial growth factor, bovine pituitary extract. Breast cancer cell lines including MCF-7, ZR-75-1, T47D, BT549, SKBR3, BT-474, MDA-MB231 and MDA-MB-361 were purchased from American Type Culture Collection and were cultured under conditions specified by the manufacturer.

The CDS sequence of C14orf166 was PCR amplified from cDNA of NBEC and cloned into the pMSCV-puro retroviral vector (indicated as C14orf166), the empty vector was used as negative control (indicated as Vector). Lipofectamine 2000 was used to transfected vectors into the indicated cells according to the manufacturer’s instruction. The same cells transfected empty vector were used as the negative control.

The C14orf166 siRNA (indicated as C14orf166/RNAi) and its cognate control siRNA (indicated as Scramble) were obtained from Guangzhou RiboBio Co (Guangdong, China). 20 nM siRNA were transfected into the indicated cells in six plates using Lipofectamine RNAiMax Reagent (Life Technologies) according to the manufacturer’s instruction.

### RNA isolation and quantitative real-time PCR

Total RNA was extracted using TRIzol reagent (Life Technologies), and cDNA was synthesized using TransScript Reverse Transcriptase (TransGen Biotech). cDNA products were amplified using CFX-96 Touch Real-Time PCR Detection System (BioRad). GAPDH were used as an internal control. mRNA levels were calculated as 2^−[(Ct of C14orf166, Cyclin D1, P21)−(Ct of GAPDH)]^, where Ct represents the threshold cycle of each transcript [16]. All experiments were performed in triplicate. The primers selected are as follows: C14orf166: forward 5′TGTTCCGACGCAAGTTGA3′ and reverse 5′CGCTGCTGTGGATGTTTCT3′.Cyclin D1:forward 5′-TCCTCTCCAAAATGCCAGAG-3′ and reverse 5′-GGCGGATTGGAAATGAACTT-3′. P21:forward 5′-CGATGCCAACCTCCTCAACGA-3′ and reverse 5′-TCGCAGACCTCCAGCATCCA-3′. GAPDH:forward 5′GGTGGTCTCCTCTGACTTC3′ and reverse 5′CTCTTCCTCTTGTGCTCTTG3′.

### Western blot and immunohistochemistry (IHC)

Cells were lysed with 50 mM Tris at pH 7.4, 150 mM NaCl, 1 mM EDTA, 1 % NP-40, 1 % TritonX-100 supplemented with protease inhibitors (Complete, Roche). The following antibodies were used: anti-C14orf166 (1:500, 19848-1-AP, Proteintech), anti-Cyclin D1 (1:1000, sc-753, Santa Cruz Biotechnology Inc), anti-P21 (1:500, sc-397, Santa Cruz Biotechnology Inc), anti-P27 (1:1000, sc-528, Santa Cruz Biotechnology Inc), anti-Rb (ab6075, Abcam) and anti-phosphorylated Rb (ab47763, Abcam). The membranes were stripped and re-probed with an anti-β-actin antibody (1:3000, HC201-02, TransGen Biotech) or anti-α-Tubulin antibody (1:1000, AT819, Beyotime) as the loading control.

IHC was performed according to standard methods as described previously [17]. The following antibodies were used: anti-C14orf166 (1:200, 19848-1-AP, Proteintech), anti-ER (1:100, ab16660, Abcam) and anti-PR (1:20, ab2765, Abcam). The results of staining were scored independently by three pathologists blinded to clinical outcome, based on both the proportion of positively stained tumors cells and the intensity of staining. The proportion of tumor cells was scored as follows: Score 0—no positive cells, Score 1—up to 10 % positive cells, Score 2—10 ~ 50 % positive cells, Score 3—50–80 % positive cells, Score 4–over 80 % positive cells. The intensity of protein expression was shown as follows: 0 (no staining), 1 (weak staining, light yellow), 2 (moderate staining, yellowish brown), and 3 (strong staining, brown).

The staining index (SI) was calculated as the product of the staining intensity and the proportion of positive cell scores (scored as 0, 1, 2, 3, 4, 6, 8, 9 or 12). Cut-off values for C14orf166 expression were chosen based on a measurement of heterogeneity using the log-rank test with respect to overall survival. The optimal cut-off was identified as an SI score of more than or equal to four was considered as having high C14orf166 expression, and a score less than 4 was considered as having C14orf166 expression.

### MTT assay

MTT assay was performed as described previously [18].

### Colony formation assay

Colony formation assay was performed as described previously [19].

### Statistical analysis

All statistical analyses were carried out by the SPSS version 19.0 software (SPSS, Chicago, IL., USA). The Chi-square test was used to analyze the correlation between C14orf166 expression and clinicopathological characteristics. Bivariate correlations between variables were calculated by Pearson’s correlation coefficient. Survival curve was plotted using Kaplan–Meier survival analysis and compared by the log-rank test. Univariate and multivariate Cox regression analyses were used to estimate the significance of various variables for survival. Results are presented as mean ± SD for at least three independent experiments, cell proliferation assay were tested using Student’s *t* test (two-tailed) for independent samples. p < 0.05 in all cases was considered significant.

## Results

### C14orf166 is upregulated in breast cancer cells and tissues

**Fig. 1:**
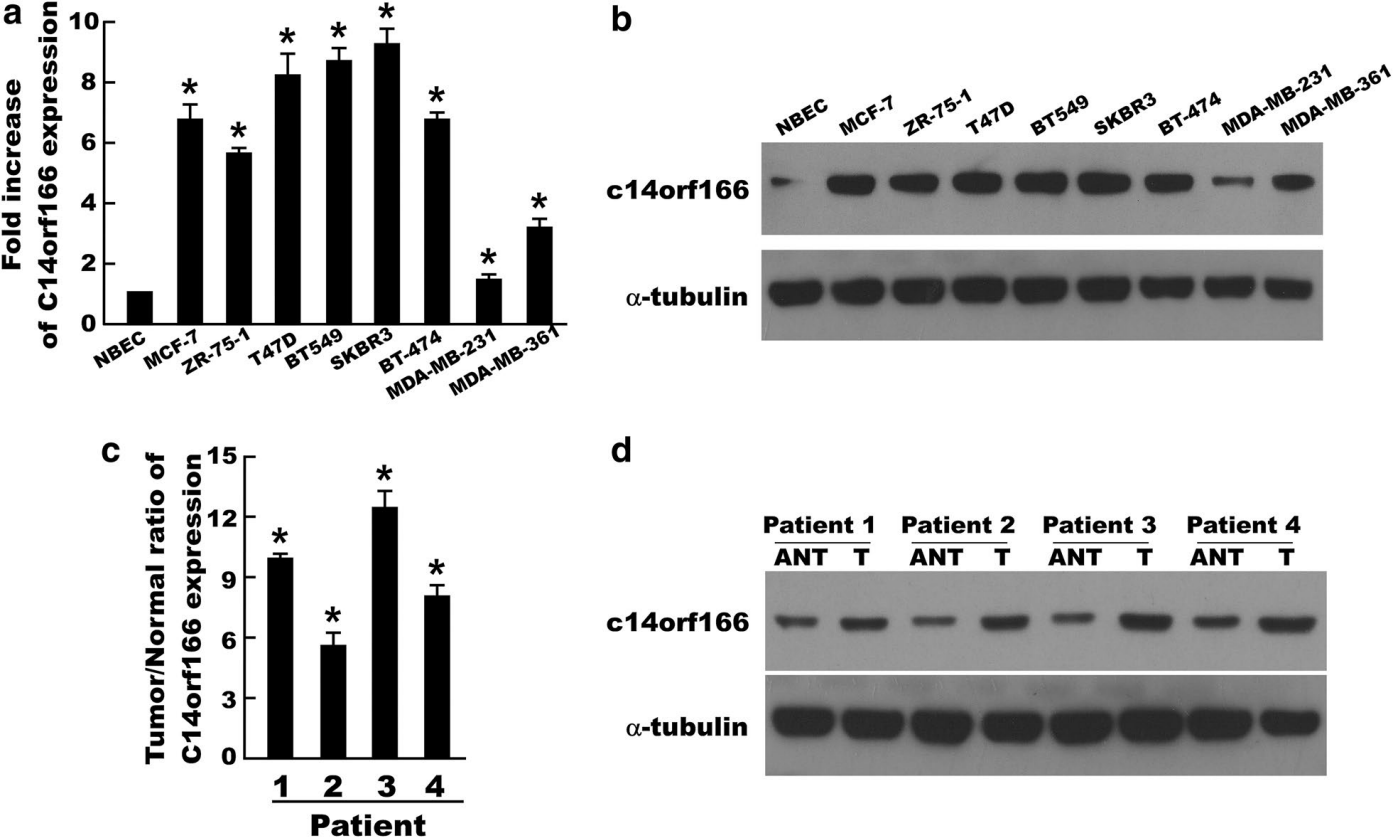
C14orf166 is upregulated in breast cancer cells and tissues. (**a**, **b**) Quantitative real-time PCR and western blot analyzed C14orf166 expression in NBEC and breast cancer cells, including MCF-7, ZR-75-1, T47D, BT549, SKBR3, BT-474, MDA-MB231 and MDA-MB-361. (**c**, **d**) Quantitative real-time PCR and western blot analyzed C14orf166 expression in four paired tumor tissues and adjacent normal tissues. *Error bars* are standard deviation of the mean (SD) calculated from three experiments performed in parallel, *p < 0.05

To investigate the role of C14orf166 in breast cancer, we first demonstrated C14orf166 expression in NBEC and breast cancer cells. Quantitative real-time PCR and western blot assay found C14orf166 expression was significantly upregulated in breast cancer cells compared to NBEC (Fig. 1a, b). To investigate whether C14orf166 was also upregulated in breast cancer tissues, we determined C14orf166 expression in four paired tumor tissues (T) and matched adjacent normal tissues (ANT), quantitative real-time PCR and western blot assay showed C14orf166 also was upregulated in breast cancer tissues compared to adjacent noncancerous tissues (Fig. 1c, d).

### C14orf166 overexpression is associated with clinical progression of breast cancer

**Fig. 2:**
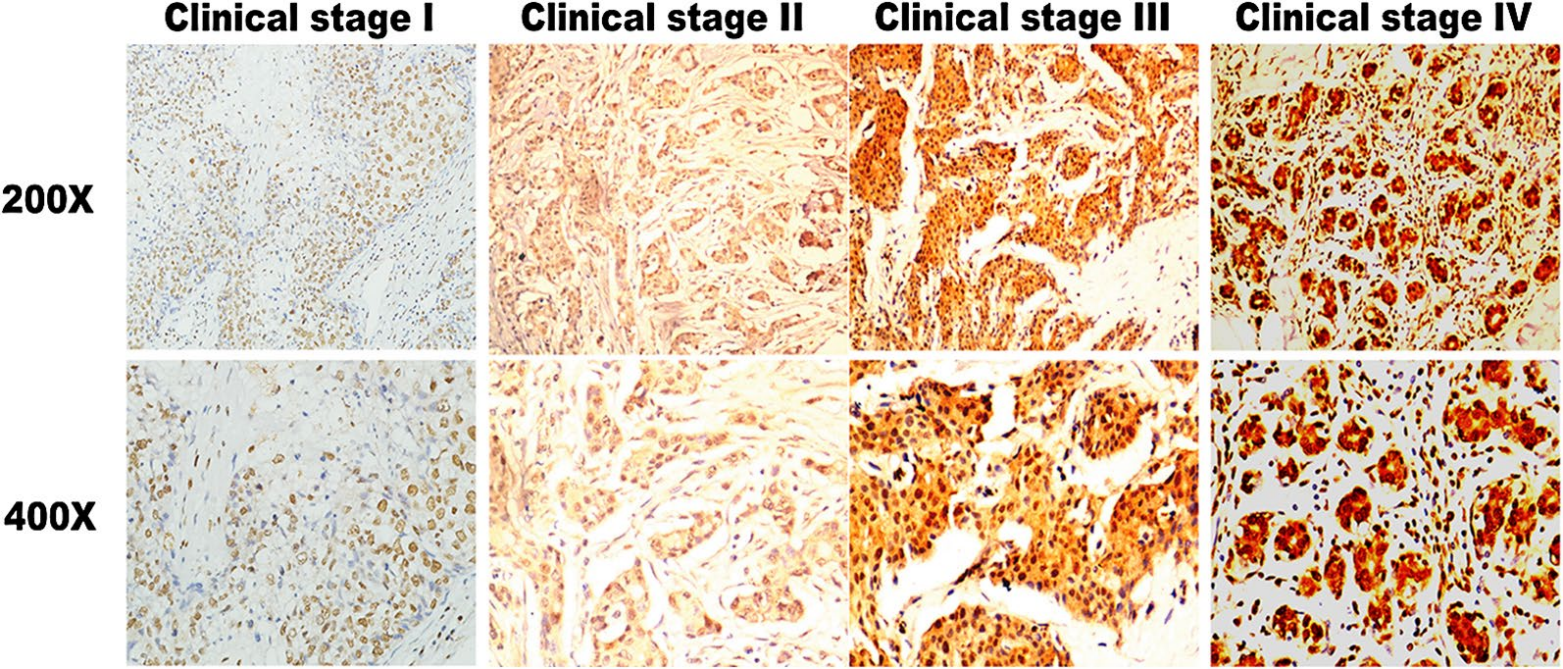
C14orf166 expression in breast cancer tissues from patients at different clinical stages. Representative images of C14orf166 expression in breast cancer tissues at different clinical stages determined by IHC

We used IHC to determine C14orf166 expression in 121 paraffin-embedded, archival breast cancer tissues, including 15 stageI tumors, 55 stage II tumors, 34 stage III tumors, 17 stage IV tumors. C14orf166 was high expression in 63 (52.1 %) cases, and no or low expression in the remaining 58 (47.9 %) cases (Table 1). The positive rate increased with advancing clinical stage as follows: 13.3 % for stage I (2/15), 43.6 % for stage II (24/55), 67.6 % for stage III (23/34), 82.4 % for stage IV (14/17) (Table 1). IHC assay shown that C14orf166 was primarily localized in the tumor cell nuclei (Fig. 2), and its expression was positively correlated with advancing clinical stage (Fig. 2), Taken together, these results suggested C14orf166 expression increased with advancing clinical stage in breast cancer.

**Table 1:** Correlation between C14orf166 expression and clinicopathologic characteristics of breast cancer patient

Characteristics		Total	C14orf166		Chi-square test *p* value
			Low expression	High expression	
Age (years)					
<40		27	12	15	0.424
≥40		94	46	48	
Clinical stage					
I		15	13	2	<0.001
II		55	31	24	
III		34	11	23	
IV		17	3	14	
T classification					
T_1_		26	17	9	<0.01
T_2_		65	34	31	
T_3_		23	6	17	
T_4_		7	1	6	
N classification					
0		45	33	21	<0.001
1		48	18	30	
2		25	6	19	
3		3	1	2	
M classification					
Yes		6	0	6	0.018
No		115	58	57	
ER					
0		51	23	28	0.325
1		58	28	30	
2		9	4	5	
3		3	3	0	
PR					
0		49	18	31	0.02
1		60	30	30	
2		10	9	1	
3		1	0	1	
4		1	1	0	
Vital status (at follow-up)					
Alive		64	51	13	<0.001
Dead		57	7	50

We examined the relationship between C14orf166 expression and clinicopathological characteristics using Chi-square test, As presented in Table 2, we revealed that high C14orf166 expression was significantly correlated with advanced clinical stage (p < 0.001), T classification (larger tumor size, p < 0.01), N classification (lymph node involvement, p < 0.001), M classification (distant metastasis, p = 0.018), PR (p = 0.02) and vital status (p < 0.001). But there were no statistically correlations between C14orf166 expression and age and ER. The correlations between C14orf166 and the clinicopathological characteristics were further confirmed used Spearman’s correlation analysis, as shown in Table 2, C14orf166 expression were significantly correlated with survival time (p < 0.001), vital status (p < 0.001), advancing clinical stage (p < 0.001), T classification (p = 0.001), N classification (p < 0.001) and PR (p = 0.009). There weren’t significance correlations between C14orf166 expression and age and ER. But distant metastasis which significantly correlated with C14orf166 expression used Chi-square test had not significance correlation with C14orf166 expression used Spearman’s correlation analysis. These results suggested C14orf166 expression was positively correlated with and advanced clinical stage, T classification, N classification, PR and vital status.

**Table 2:** Spearman analysis of correlation between C14orf166 and clinicopathological characteristics

Variables	C14orf166 expression level	
	Spearman correlation	*p* Value
Survival time	−0.735	<0.001
Vital status	0.674	<0.001
Age	−0.037	0.684
ER	−0.068	0.280
PR	−0.236	0.009
Clinical stage	0.404	<0.001
T classification	0.295	0.001
N classification	0.390	<0.001
M classification	0.088	0.270

### C14orf166 is a poor prognostic factor for patients with breast cancer

**Fig. 3:**
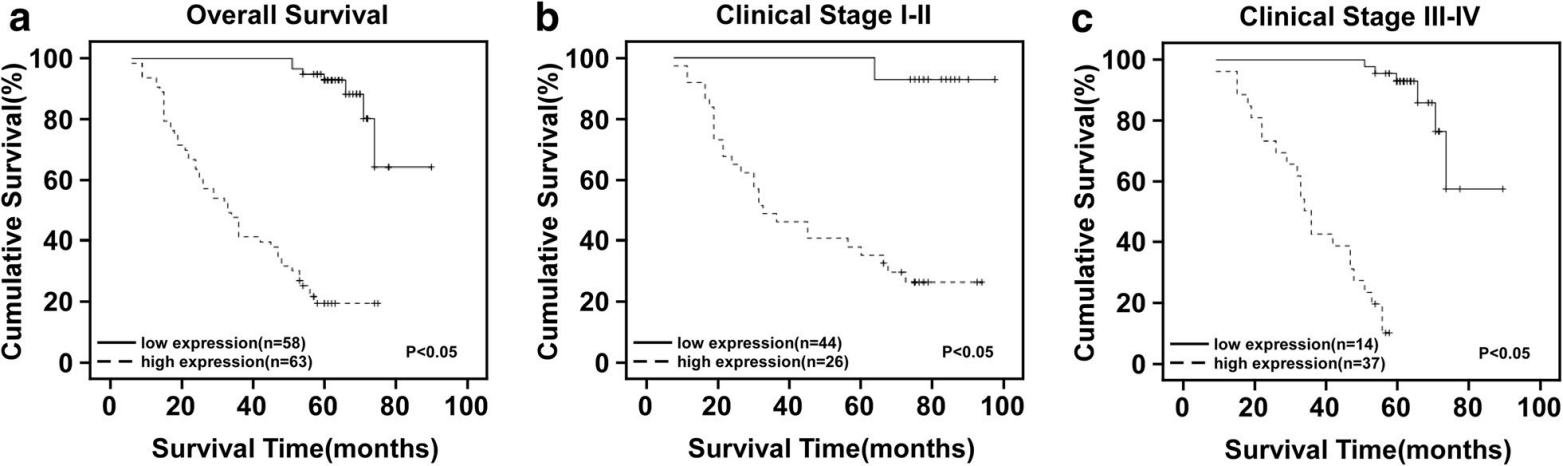
The relationship between C14orf166 expression and clinical outcome. **a** Kaplan–Meier overall survival curves for the patients with high versus low C14orf166 expression. **b** Kaplan–Meier overall survival curves for the patients at clinical stage I–II with high versus low C14orf166 expression. **c** Kaplan–Meier overall survival curves for the patients at clinical stage III–IV with high versus low C14orf166 expression

Kaplan–Meier survival analysis and the log-rank test demonstrated that the overall survival of the patients with high C14orf166 expression was significantly shorter than those with low C14orf166 expression (Fig. 3a). Univariate Cox regression analysis showed that C14orf166 expression (p < 0.001), clinical stage (p = 0.013), T classification (p < 0.001) and N classification (p = 0.007) were unfavorable prognostic factors (Table 3). To determine whether C14orf166 expression is an independent prognostic factor of outcomes, multivariate survival analysis revealed C14orf166 expression, clinical stage, T classification and N classification were recognized as independent prognostic factors for patients with breast cancer (Table 3).

**Table 3:** Univariate and multivariate analyses of various prognostic parameters in patients with liver cancer Cox-regression analysis

	Univariate analysis			Multivariate analysis		
	No. patients	*p*	Regression coefficient (SE)	*p*	Relative risk	95 % Confidence interval
C14orf166		<0.001	2.678 (0.412)	<0.001	15.231	6.472–35.847
Low expression	58					
High expression	63					
Clinical stage		0.013	0.363 (0.145)	0.044	0.702	0.498–0.990
I	15					
II	55					
III	37					
IV	17					
T classification		<0.001	0.623 (0.170)	0.007	1.705	1.156–2.516
T_1_	26					
T_2_ T_3_	65					
T_3_	23					
T_4_	7					
N classification		0.007	0.389 (0.145)	0.001	4.598	1.878–11.256
0	45					
1	48					
2	25					
3	3					

Additionally, we analyzed the prognostic value of C14orf166 expression in selective subgroups of breast cancer patients stratified according to clinical stage. Patients with tumors exhibiting high C14orf166 expression had significantly shorter overall survival than those with low C14orf166 expression in the clinical stage I–II subgroup (Fig. 3b, p < 0.05) and clinical stage III–IV subgroup (Fig. 3c, p < 0.05). These suggested C14orf166 was a valuable prognostic factor for patients with breast cancer.

### C14orf166 contributes to cell proliferation of breast cancer

**Fig. 4:**
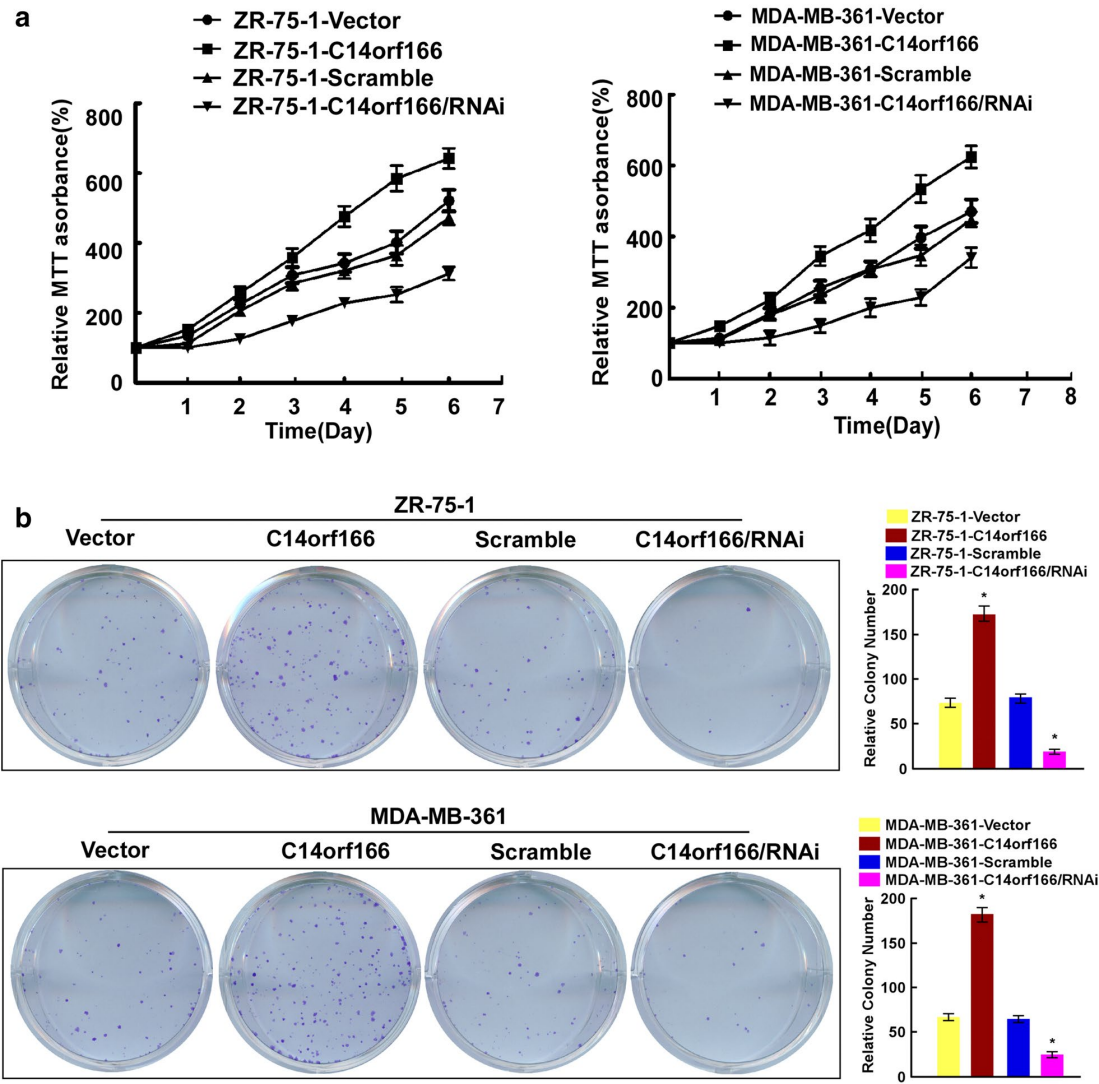
C14orf166 contributes to the proliferation of breast cancer. **a** MTT assay investigated the role of C14orf166 expression in cell proliferation by modulating its expression in breast cancer cells ZR-75-1 and MDA-MB-361. **b** Colony formation assay determined the role of C14orf166 expression in cell proliferation. Representative micrographs (*left*) and quantification (*right*) of crystal violent stained cell colonies. *Error bars* are standard deviation of the mean (SD) calculated from three experiments performed in parallel, *p < 0.05

To explore the role of C14orf166 in the progression of breast cancer cells, we overexpressed C14or166 in breast cancer cells ZR-75-1 and MDA-MB361 to determine its effect on cell proliferation, MTT analysis found C14orf166 overexpression increased proliferation rate of breast cancer cells (Fig. 4a), colony formation assay suggested C14orf166 overexpression significantly promoted proliferation of breast cancer cells (Fig. 4b). To further examined the effect of C14orff166 on cell proliferation, we downregulated C14orf166 used small inference RNA (siRNA), MTT analysis found knockdown of C14orf166 reduced proliferation rate of breast cancer cells (Fig. 4a), colony formation assay confirmed that C14orf166 knockdown significantly inhibited proliferation of breast cancer cells (Fig. 4b). Taken together, these results suggested C14orf166 regulated the proliferation of breast cancer cells.

**Fig. 5:**
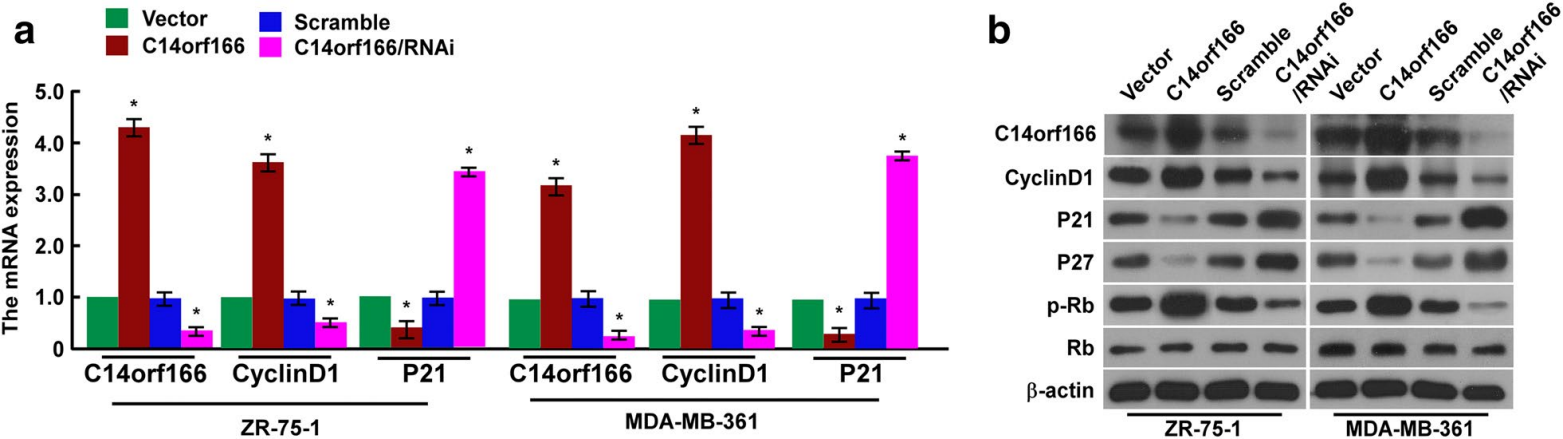
C14orf166 regulates the expression of key cell cycle proteins. **a** Quantitative real-time PCR determined P21 and Cyclin D1 expression by modulating its expression in indicated breast cancer cells. **b** Western blot analyzed Cyclin D1, P21, P27, p-Rb and Rb expression by modulating its expression in indicated breast cancer cells. *Error bars* are standard deviation of the mean (SD) calculated from three experiments performed in parallel, *p < 0.05

Cell proliferation is regulated by many key cell cycle regulatory proteins, including P21, P27, Cyclin D1, Rb and the phosphorylation level of Rb. These proteins play critical role in G1/S transition. We investigated their levels by modulating C14orf166 expression. Quantitative real-time PCR assay found when C14orf166 was overexpressed in indicated breast cancer cells, P21 and P27 were upregulated, when C14orf166 was downregulated, P12 and P27 were downregulated (Fig. 5a). Western blot showed that Cyclin D1 and the phosphorylation level of Rb were decreased, P21 and P27 were increased, Rb was not changed, when C14orf166 was overexpressed. Cyclin D1 and the phosphorylation level of Rb were increased, P21 and P27 were decreased, when C14orf166 was downregulated (Fig. 5b). These suggested C14orf166 promoted cell proliferation by accelerating G1/S transition. We have found C14orf166 expression was correlated with T classification, the results investigated from breast cancer cells also found C14orf166 promoted cell proliferation. Results from clinic and cells were consistent.

## Discussion

In our current studies, we revealed C14orf166 was overexpressed in breast cancer cells and tissues, it might be a novel oncogene, we further analyzed C14orf166 expression in a cohort of 121 samples by IHC, and investigated the correlation between C14orf166 expression and clinicopathological characteristics. Statistical analyses suggested C14orf166 expression was increased with advancing clinical stage, and had significance correlation with advanced clinical stage, T classification, N classification, PR and vital status. Patients with high C14orf166 expression had shorter survival than those with low C14orf166 expression. C14orf166 could serve as a valuable prognostic factor. We also determined the role of C14orf166 in cellular proliferation, and found overexpression of C14orf166 promoted cellular proliferation, knockdown of C14orf166 inhibited cellular proliferation. Mechanism analysis revealed C14orf166 promoted cell proliferation by accelerating G1/S transition.

Centrosomal protein ninein interacts with glycogen synthase kinase 3beta (GSK-3β), and be phosphorylated by GSK-3β [20]. The activity of Janus kinase 2 (JAK2) is inhibited by ninein [21], constitutive activation of JAK2/STAT5 promotes resistance to apoptosis and contributes to tumorigenesis of breast cancer [22], these suggest ninein may inhibit the development and progression of breast cancer. C14orf166 interacts with ninein to block the phosphorylation of ninein catalyzed by GSK-3β [13], suggesting C14orf166 may inhibit ninein, and activate JAK2 to promote breast cancer progression, but this speculation remain to be confirmation. The regulation mechanism of C14orf166 is still to be explored. JAK2/STAT3 promotes self-renewal of breast cancer stem cells, CD24^−^CD44^+^ is a marker for breast cancer stem cells [23, 24]. Activation of JAK2/STAT3 pathway preferentially promotes the self-renewal of C24^−^CD44^+^ breast cancer stem cells [25]. It reveals that C14orf166 may promote self-renewal of breast cancer stem cells.

Yazhou Cui et, al. find C14orf166 is higher expression in pancreatic cancer with lymph node metastasis than that of in pancreatic cancer without lymph node metastasis by difference gel electrophoresis, they speculate C14orf166 could be a novel metastasis-associated protein [26]. In our study, we found there was a correlation between C14orf166 expression and lymph node metastasis, it was aggressed with previous report. But there wasn’t a correlation between C14orf166 expression and distant metastasis. The different results may cause by different kinds of tumors. However, our findings still need to be replicated, and further investigation used more patients’ tissues is required to verify these hypotheses.

## Conclusions

In summary, our research show that C14orf166 is an unfavorable prognostic factor for breast cancer patients, patients with high C14orf166 expression have shorter survival than those with low C14orf166 expression. C14orf166 accelerates G1/S transition and promotes proliferation of breast cancer cell.

## Additional file

10.1186/s12967-016-0805-0 Clinicopathological characteristics of patient samples and expression of C14orf166 in breast cancer.
