# Supplementary material for: C14orf166 overexpression correlates with tumor progression and poor prognosis of breast cancer
Source: J Transl Med. 2016 Feb 17;14:54. doi: 10.1186/s12967-016-0805-0 (PMC4756411; doi:10.1186/s12967-016-0805-0)
Supplement: Supplementary file 1 — 10.1186/s12967-016-0805-0 Clinicopathological characteristics of patient samples and expression of C14orf166 in breast cancer. [file 12967_2016_805_MOESM1_ESM.docx]

*Table* *1.* Clinicopathological characteristics of patient samples and expression of C14orf166 in breast cancer

| **Characteristics** | Number of cases (%) |
| --- | --- |
| **Age (years)** |  |
| <40 | 27（22.3） |
| ≥40 | 94（77.7） |
| **Gender** |  |
| Male | 0(0) |
| Female | 121(100) |
| **Clinical stage** |  |
| I | 15（12.4） |
| II | 55（45.5） |
| III | 34（28.1） |
| IV | 17（14） |
| **T classification** |  |
| T_1_ | 26（21.4） |
| T_2_ | 65（53.7） |
| T_3_ | 23（19.0） |
| T_4_ | 7（5.9） |
| **N classification** |  |
| N_0_ | 45（37.2） |
| N_1_ | 48（39.7） |
| N_2_ | 25(20.7) |
| N_3_ | 3(2.4) |
| **M classification** |  |
| No | 115（95.0） |
| Yes | 6（5.0） |
| **Vital status (at follow-up)** |  |
| alive | 64（52.9） |
| Dead | 57（47.1） |
| **Expression of C14orf166** |  |
| Low expression | 58（47.9） |
| High expression | 63（52.1） |
| **ER** |  |
| 0 | 51（42.2） |
| 1 | 58（47.9） |
| 2 | 9(7.5) |
| 3 | 3(2.4) |
| **PR** |  |
| 0 | 49（40.5） |
| 1 | 60（49.6） |
| 2 | 10(8.3) |
| 3 | 1(0.8) |
| 4 | 1(0.8) |
